# Addressing antimicrobial resistance: Current challenges, emerging strategies, and an AI-powered, community-driven approach

**DOI:** 10.1093/pnasnexus/pgag273

**Published:** 2026-08-11

**Authors:** Peter Wang, Alton Hsiung, Christiana Fraise, Gyula Seres, Li Ming Chong, Kui You, Angelina Moh, Chengxun Su, Yoann Sapanel, Isaiah Zhuang, Lissa Hooi, Oon Tek Ng, Shawn Vasoo, Dean Ho

**Affiliations:** Department of Biomedical Engineering, College of Design and Engineering, National University of Singapore, Singapore 117583; Institute for Digital Medicine (WisDM), Yong Loo Lin School of Medicine, National University of Singapore, Singapore 117456; The N.1 Institute for Health (N.1), National University of Singapore, Singapore 117456; Institute for Digital Medicine (WisDM), Yong Loo Lin School of Medicine, National University of Singapore, Singapore 117456; The N.1 Institute for Health (N.1), National University of Singapore, Singapore 117456; Institute for Digital Medicine (WisDM), Yong Loo Lin School of Medicine, National University of Singapore, Singapore 117456; The N.1 Institute for Health (N.1), National University of Singapore, Singapore 117456; Institute for Digital Medicine (WisDM), Yong Loo Lin School of Medicine, National University of Singapore, Singapore 117456; The N.1 Institute for Health (N.1), National University of Singapore, Singapore 117456; Department of Biomedical Engineering, College of Design and Engineering, National University of Singapore, Singapore 117583; Institute for Digital Medicine (WisDM), Yong Loo Lin School of Medicine, National University of Singapore, Singapore 117456; The N.1 Institute for Health (N.1), National University of Singapore, Singapore 117456; Department of Biomedical Engineering, College of Design and Engineering, National University of Singapore, Singapore 117583; Institute for Digital Medicine (WisDM), Yong Loo Lin School of Medicine, National University of Singapore, Singapore 117456; The N.1 Institute for Health (N.1), National University of Singapore, Singapore 117456; Institute for Digital Medicine (WisDM), Yong Loo Lin School of Medicine, National University of Singapore, Singapore 117456; The N.1 Institute for Health (N.1), National University of Singapore, Singapore 117456; Institute for Digital Medicine (WisDM), Yong Loo Lin School of Medicine, National University of Singapore, Singapore 117456; The N.1 Institute for Health (N.1), National University of Singapore, Singapore 117456; Institute for Digital Medicine (WisDM), Yong Loo Lin School of Medicine, National University of Singapore, Singapore 117456; The N.1 Institute for Health (N.1), National University of Singapore, Singapore 117456; Institute for Digital Medicine (WisDM), Yong Loo Lin School of Medicine, National University of Singapore, Singapore 117456; The N.1 Institute for Health (N.1), National University of Singapore, Singapore 117456; Department of Pharmacology, Yong Loo Lin School of Medicine, National University of Singapore, Singapore 117600; Department of Biomedical Engineering, College of Design and Engineering, National University of Singapore, Singapore 117583; Institute for Digital Medicine (WisDM), Yong Loo Lin School of Medicine, National University of Singapore, Singapore 117456; The N.1 Institute for Health (N.1), National University of Singapore, Singapore 117456; National Centre for Infectious Diseases (NCID), Singapore 308442; National Centre for Infectious Diseases (NCID), Singapore 308442; Department of Biomedical Engineering, College of Design and Engineering, National University of Singapore, Singapore 117583; Institute for Digital Medicine (WisDM), Yong Loo Lin School of Medicine, National University of Singapore, Singapore 117456; The N.1 Institute for Health (N.1), National University of Singapore, Singapore 117456; Department of Pharmacology, Yong Loo Lin School of Medicine, National University of Singapore, Singapore 117600; The Bia-Echo Asia Centre for Reproductive Longevity and Equality (ACRLE), National University of Singapore, Singapore 117456

**Keywords:** antimicrobial resistance, antibiotics, artificial intelligence, governance, public health

## Abstract

The rising incidence of antimicrobial resistance (AMR) has threatened global public health with a high mortality rate. In parallel, the de-incentivization for further investment, regulatory constraints, and overuse/misuse of antibiotics have accelerated the progression of AMR. Together, these factors have contributed to the declining efficacy of a wide spectrum of antibiotics and the increasing prevalence of drug resistance that outpaced AMR-specific drug development. More importantly, even with substantial historical investments in antibiotic discovery over several decades, the development of clinically actionable, novel antibiotics has remained limited. In this article, we present the challenges of addressing AMR and outline emerging strategies that may reduce its burden. While advanced therapies, including bacteriophage therapy, have demonstrated promising results, complementary strategies that integrate emerging technologies and engage diverse stakeholders are needed. Building on this vision, we propose the potential role of AI-powered platforms in supporting and accelerating AMR-specific drug development alongside a community-driven approach that engages scientists, policymakers, health economists, clinicians, and patients to help address existing scientific and translational challenges. Furthermore, this article offers an inclusive strategy with key considerations, including educational and public health interventions, government-led programs, and health economics, which together could potentially tackle the threat of AMR along with AI-powered solutions.

## Introduction

Antimicrobial resistance (AMR) is a major global health crisis, rendering once-effective treatments ineffective against emerging bacterial infections; it is expected to kill as many patients as cancers in the coming decades (1–5). According to the World Health Organization (WHO) global antibiotic resistance surveillance report, ∼40% of monitored pathogen–antibiotic pairs between 2018 and 2023 exhibited resistance (6). Consistent with the trend, the resistance rate of levofloxacin in the Asia Pacific region has increased from 2 to 27% in the last two decades (7). Notably, Southeast Asia and the Eastern Mediterranean regions have the highest observed prevalence of antibiotic resistance with approximately one in three laboratory-confirmed infections caused by antibiotic-resistant pathogens (6). This resistance occurs when organisms evolve to withstand antimicrobial treatments. AMR arises through genetic mutations and horizontal gene transfer. The overuse of antibiotics, incomplete treatment courses, environmental contamination, climate change, and food-animal production generate selective pressures that further amplify the emergence of AMR (8–10). However, the overuse and misuse of antibiotics are the most important factors leading to the escalating incidences of AMR (8). The consequences of AMR extend beyond individual health, posing risks to modern medicine. Routine surgeries, cancer treatments, and organ transplants depend on effective antibiotics to prevent infections (11, 12). To address the emergence of AMR, Louis B. Rice first coined a list of highly virulent and resistant pathogens, known as the “ESKAPE” pathogens, consisting of *Enterococcus faecium*, *Staphylococcus aureus*, *Klebsiella pneumoniae*, *Acinetobacter baumannii*, *Pseudomonas aeruginosa*, and *Enterobacter* sp. in 2008 (13). The ESKAPE term was subsequently adopted by WHO for these pathogens. However, even with substantial historical investments into antibiotic discovery and development, pharmaceutical companies have increasingly deprioritized antibiotic development over the past few decades. This may be attributed to the limited discovery of additional antibiotic classes, driven not only by low financial returns but also by scientific bottlenecks and the lack of major breakthroughs (14, 15). Therefore, simultaneously raising public health awareness of proper antibiotic usage and harnessing other sustainable approaches to develop AMR-specific treatment strategies are critical to overcoming this arms race against emerging bacterial resistance and mutations.

Understanding antimicrobial use in clinician–patient engagement may substantially prevent inappropriate use (8, 16). Overprescribing antibiotics is a particular concern in primary care, where 90% of antibiotics are prescribed by general practitioners (17). These antibiotics are mostly prescribed for respiratory infections (17). Implementing government-led stewardship programs that oversee antibiotic use may ensure institutional compliance. Aside from clinicians, educating patients on the proper usage of antibiotics is also critically important. Instead of informing patients of the guidelines when prescribing antibiotics, broadly raising public health awareness to understand antimicrobial use may improve awareness in the clinician–patient pair. Alongside educational interventions, effectively communicating the threat posed by AMR to both medical professionals and the public may alleviate the primary challenge leading to AMR.

The integration of AI-powered solutions with clinical workflows may lead to enhancement in treatment outcomes (18–21). Notably, unlike earlier eras of antibiotic discovery, the modern drug development approach typically requires 10+ years for the commercialization of the therapeutic (3–5, 19–22). However, the timeline for drug development is outpaced by the mutation rate of pathogens. More importantly, by the time a targeted therapy is developed, the pathogen may have already mutated. Therefore, a rapid, actionable, and sustainable approach to developing effective antibiotics and/or AMR-specific treatment strategies represents a strategic tool for future pandemic readiness. Developing treatments with AI-powered solutions has laid important groundwork to combat the pathogens in the long-lasting arms race (23). These AI-driven strategies may provide important alternatives to traditional drug development approaches, leading to more sustainable options in combatting AMR. Furthermore, alongside AI-powered solutions, an inclusive and community-driven effort may actionably and sustainably address the challenges posed by the emergence of AMR. Moving away from studying drug discovery and policy in isolation, this article critically evaluates the plausible role of AI across multiple stages of antibiotic development. Therefore, this article acknowledges both the potential and current limitations of AI and assesses the technological considerations within an integrated, community-driven framework spanning governance, health economics, and public health interventions.

## Why is AMR a concern?

AMR poses a growing global threat to public health, with increasingly dire implications for both individual treatment outcomes and the broader healthcare system. According to WHO, AMR is responsible for an estimated 1.27 million deaths globally each year, with projections suggesting this number could rise to 10 million annually by 2050, if no action is taken (1, 2, 8). The consequences of AMR extend beyond mortality; resistant infections lead to prolonged hospital stays, increased medical costs, and heightened risks during common procedures, such as surgery, chemotherapy, and childbirth (24). Resistant strains of *Escherichia coli*, *K. pneumoniae*, *S. aureus*, and *A. baumannii* are particularly concerning, often displaying resistance to multiple antibiotic classes. For example, in Japan, an ecological study analyzing data from 2015 to 2016 reported levofloxacin resistance rates in *E. coli* ranging from 26.2 to 55.0% across prefectures (25), with higher resistance observed in Western regions. Additionally, the resistance rate of levofloxacin in the Asia Pacific region has increased from 2 to 27% in the last two decades (7). This sharp rise reflects a broader regional trend in Asia Pacific, where fluoroquinolone resistance has escalated significantly over the past two decades. Similarly, carbapenem resistance in *K. pneumoniae* has surpassed 50% in some regions, rendering many last-resort antibiotics ineffective (26).

The misuse and overuse of antibiotics in healthcare and agriculture remain primary drivers of AMR. In some countries, up to 80% of medically important antibiotics are used in food-producing animals, often for growth promotion or disease prevention rather than treatment (27). Increasingly, environmental factors, especially climate change and pollution, are being recognized as key accelerators of resistance evolution and spread. Rising global temperatures have been linked to increased bacterial growth rates and enhanced rates of horizontal gene transfers. Warmer climates promote longer survival of pathogens in water and soil. According to a 2018 study, every 10 °C difference across regions in local minimum temperature was associated with a 4.2% increase in antibiotic resistance in *E. coli*, 2.2% in *K. pneumoniae*, and 2.7% in *S. aureus* (28).

## Current trends in developing antibiotics

Developing new antibiotics through drug discovery and development is one of the most direct approaches to combating AMR. However, this process presents scientific challenges. Even though newly developed antibiotics may provide additional treatment options, successful strategies for discovering novel antibiotics should also prioritize the selection of chemical entities with reduced potential for resistance emergence, instead of focusing solely on identifying compounds with (i) novel mechanisms of action and (ii) reduced cross-resistance to existing antibiotics. In some cases, new antibiotics may also provide solutions for persistent infections that are no longer treatable with existing drugs. However, developing novel antibiotics is a time-consuming and expensive process. The modern drug development timeline takes ∼10–15 years to progress from initial discovery to regulatory approval (22). This timeline reflects both stricter comprehensive regulatory pathways and limited discoveries of novel antibacterial agents. Given these obstacles, there is increasing interest in alternative, complementary strategies, including drug repurposing and AI-driven drug development, which may improve target identification, screening efficiency, and other parts of the workflow. Nonetheless, AI-enabled tools for antibiotic discovery and development may play an important role in improving these workflows toward more sustainable, cost-effective, and efficient strategies for addressing AMR.

### Challenges in developing antibiotics

Though designing a targeted therapy for a given indication has various advantages, it is important to realize that the time required to develop a new drug is significantly longer than the time pathogens mutate and develop resistance (29). The typical timeline to develop a new drug is now 10–15 years (22). For example, the development of meropenem, one of the most important antibiotics that has broad-spectrum activities against various pathogens, took about 13 years (30, 31). In the case of the COVID-19 pandemic, the SARS-CoV-2 virus has already mutated from beta to omicron variants, including sub-variants (32). Moreover, even with substantial historical investments into antibiotic development, most antibiotics developed after 1980 have mostly been derived from existing therapies, and thus, there have not been any new classes of antibiotics (33, 34). Furthermore, the drug development community has mostly focused on single-enzyme target discovery, which may be vulnerable to resistance arising from single-step mutations. While such approaches have led to multiple clinically actionable antibiotics, reliance on single-target strategies may limit long-term actionability against resistance. Altogether, developing novel antibiotics remains highly challenging.

### Regulatory constraints

**Figure 1:**
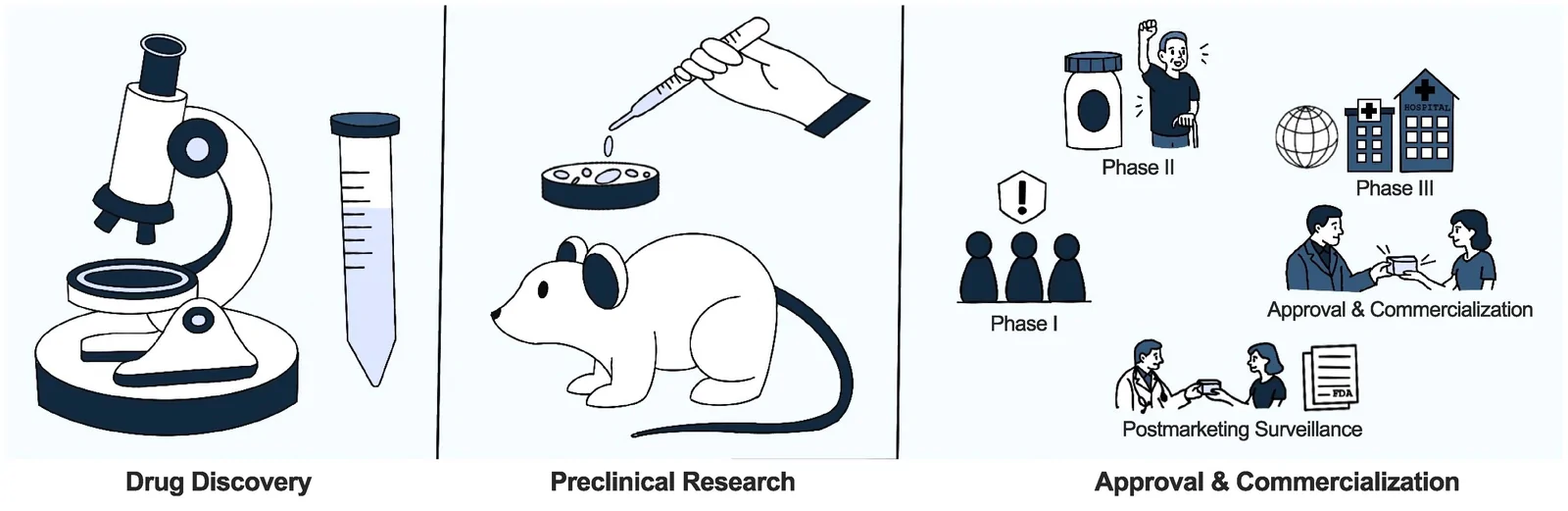
Drug development workflow. The conventional workflow begins with drug discovery experiments that aim to identify drug candidates (e.g. target identification). Subsequently, the candidate is validated in various preclinical models to evaluate safety and activity against a given indication. With promising preclinical data, a drug candidate may proceed to request human trials from regulatory agencies (e.g. FDA) with multiple stages of clinical trials prior to commercialization. Typically, postmarketing surveillance from FDA continues to monitor the risk of the approved drug.

The competition between drug development and drug resistance is further exacerbated by the minimal incentive of pharmaceutical companies to invest in new antibiotic development, following decades of investments into the field. The lack of incentive can be attributed to: (i) low regulatory approval rate, (ii) limited uptake of antibiotics under certain government-intervened programs, and (iii) low benefit–cost ratio. Developing a new drug typically involves complex phases leading to bench-to-bedside translation. The very initial steps require comprehensive drug discovery followed by preclinical studies. The drug candidate subsequently undergoes clinical trials: phase I, phase II, and phase III. With these clinical outcomes, the therapeutic will be comprehensively assessed via regulatory agencies (e.g. Food and Drug Administration [FDA]) for eventual commercialization (Fig. 1). Due to this lengthy and rigorous workflow, the regulatory approval rate of newly developed antibiotics is low (33–35). For instance, according to a WHO report, only 12 antibiotics have been approved since 2017 (36). Most of these drugs are derived from existing classes of antibiotics. More importantly, only one in 15 antibiotics will reach a patient and one in 30 antibiotics of new classes will eventually be administered for infections (33, 34, 36). In a report from the United States Center for Disease Control and Prevention (CDC), ∼30% of antibiotics prescribed in the United States are considered unnecessary (37). This same trend is observed globally and as a result, stewardship programs have been established to monitor and improve the prescription of antibiotics. For instance, the antimicrobial stewardship programs (ASPs) funded by the CDC have been employed to improve antibiotic usage country-wide (38, 39). Additionally, expanded expertise on proper usage along with limited antibiotic uptake to reduce potential resistance has substantially reduced profits and returns for pharmaceutical companies, pointing investors toward other high-profit investments in drug development, especially in cancer therapies (33, 34).

### Cost–benefit considerations

Furthermore, the low benefit–cost ratio in the drug development of antibiotics contributes to the deprioritization of pharmaceutical companies. One of the major challenges in addressing AMR is the economic disincentive for pharmaceutical companies to invest in new antibiotic development, even with large-scale investments in the past few decades. Unlike medications for chronic diseases, which generate long-term profit because of continuous patient use, antibiotics are usually taken for short durations and are often reserved for extreme cases. The estimated cost of developing a new antibiotic is ∼$1.5 billion (40). Moreover, as patents of existing antibiotics are expiring, more companies are manufacturing antibiotics, leading to more competition and lower prices for medical use (34). With the above-mentioned factors, the annual revenue of an antibiotic is ∼$46 million, which is not sufficient to justify further investments into the discovery of new antibiotics. Altogether, these factors limit the market potential of new antibiotics and discourage companies from investing in research and development (R&D). The high costs associated with bringing a new antibiotic to market, coupled with rigorous regulatory requirements, make the financial risk of antibiotic development unappealing. The development of new antibiotics requires more incentive as well as government-led policies to drive more investment in antibiotics. While this issue may not be resolved in a short period of time, alternative strategies, including advanced therapeutics and AI-powered solutions, may help combat AMR.

## Emerging alternative advanced therapies

Antibiotics are the primary treatment option in the era of AMR. However, alternative advanced therapies are emerging and have provided clinical teams with various options to explore in this race against bacterial resistance. For example, recently in Singapore, a female patient who had undergone heart surgery was infected by *P. aeruginosa* in her chest cavity and blood (41). In searching for host-specific phages, the clinical team examined their catalogs and the environment in Singapore. The team found an effective cocktail consisting of antibiotics and three phages including one obtained from the Singapore River. The patient later avoided a high-risk surgery. This case represents an example of how alternative therapies including monoclonal antibodies (mAbs), antimicrobial peptides (AMPs), and nanoparticles may be integrated into the existing clinical workflows for managing bacterial infections (Table 1). Critically, the success of these alternative strategies especially their efficacy and safety may require further validation. Findings derived from in vitro studies do not necessarily translate to in vivo and clinical efficacy. Nonetheless, while these alternative strategies may not be broadly applicable, they may still provide complementary treatment options to the clinical teams, similar to the Singapore River–derived bacteriophage therapy.

**Table 1:** Clinical trials of alternative treatment strategies against AMR.

AMR treatment strategies	NCT (ClinicalTrials.gov)	Status
Bacteriophage	NCT00945087	Unknown
	NCT05498363	Completed
Monoclonal antibodies	NCT06621251	Recruiting
	NCT04335383	Recruiting
	NCT05355207	Unknown
	NCT02508584	Completed
Antimicrobial peptides	NCT05340790	Recruiting
	NCT00563394	Completed
	NCT00563433	Completed
	NCT02849262	Completed
	NCT02596074	Completed
CRISPR-Cas system	NCT05488340	Recruiting
Nanoparticles	NCT05268718	Unknown
	NCT03752424	Unknown
	NCT04775238	Unknown
	NCT04481945	Completed
	NCT01950546	Completed

A list of relevant clinical trials on ClinicalTrials.gov (NCTs) is summarized in the table.

### Bacteriophage therapy

Phage therapy has seen a resurgence in interest due to the potential it has as a self-replicating solution for AMR. Infections are treated by using viruses to eliminate bacteria. Phage therapy is host specific (41, 42), which leads to precise and specific targeting of the bacteria (host). However, as this approach is specific, it requires time to determine suitable phages for a given species of bacteria. Recent studies have harnessed genome engineering to customize phages for different indications (43–46). Community-driven efforts have been put into developing phage banks that store bacterial strains and phages for broader clinical translation. For example, the University of California, San Diego (UC San Diego) has established the Center for Innovative Phage Applications and Therapeutics (47). Similarly, in Belgium, the Queen Astrid Military Hospital is hosting a phage bank containing samples and matching phages (48). However, a limitation of phage therapy is the ability of bacteria to quickly evolve phage resistance (49, 50). This necessitates the use of combination strategies, such as pairing phages with antibiotics to maintain therapeutic efficiency. Despite these challenges, trials have demonstrated the potential of phage therapy (Table 1).

Currently, bacteriophages are administered for patients experiencing life-threatening conditions who have exhausted all possible treatments (51). Bacteriophage-based therapies have improved clinical outcomes for infected patients. In a cohort of nine subjects with cystic fibrosis, personalized inhaled bacteriophage therapies were administered based on the susceptibility of *P. aeruginosa* sputum isolates. This therapy resulted in decreased bacteria in sputum, improved lung function, and reduced bacterial virulence (52). Similarly, another study reported the use of a personalized bacteriophage-based treatment for a 62-y-old diabetic patient with pancreatitis complicated by multidrug-resistance *A. baumannii* (53). The patient experienced a downward clinical trajectory after failing multiple antibiotics in a 4-month period. Nine phages were identified and administering three of them resulted in improved clinical outcomes. Recently, a patient at the University of Pittsburgh Health Sciences infected by *En. faecium* with exhausted antibiotic-based treatments was successfully treated by bacteriophages isolated from wastewater (54). The success of bacteriophages in overcoming highly resistant bacterial infections is evident (42, 55–57). Continuous efforts to diversify the arsenals against AMR with phage-based therapies and/or phage-centric combination therapies with antibiotics are necessary to overcome AMR challenges (58).

### Monoclonal antibodies

mAbs are a class of antibodies that are uniform, and they can bind to target antigens. mAbs may be used to combat bacterial AMR in several ways: (i) neutralize bacterial toxins to reduce the resulted damage; (ii) bind with bacteria to form complexes that may be cleared by the innate immune system; and (iii) elicit bacterial cell lysis. Due to their high specificity, mAbs have minimal off-target effects, leaving beneficial microbes unaffected.

mAbs have success in constraining resistant bacteria. In a recent study, Baker et al. identified mAb 1416 capable of inhibiting carbapenem-resistant *A. baumannii* associated with neonatal sepsis deaths (59). Mice treated with mAb 1416 showed a reduction in bacterial load compared with controls, demonstrating the potential of this mAb to protect neonates from sepsis. Given their potency against bacteria, some mAbs have gone through clinical trials and gained approval from the US FDA. For instance, raxibacumab was approved in 2012 to treat inhalational anthrax following *Bacillus anthracis* infection (60), and bezlotoxumab was approved in 2016, though later discontinued in early 2025, for diarrhea caused by *Clostridium difficile* (61). Furthermore, panobacumab, which is not yet FDA approved, promotes complement-mediated opsonophagocytic clearance of *P. aeruginosa*. In a phase IIa trial for nosocomial pneumonia, it demonstrates improved clinical outcomes in a shorter time when used as a complementary treatment (62).

Despite their potential against AMR, mAbs may need to address challenges before achieving clinical success and large-scale implementation into existing clinical protocols (63). First, high specificity renders mAbs effective against only pathogens with the targeted antigens, thus providing narrower coverage compared with traditional broad-spectrum antimicrobials due to antigenic heterogeneity. Second, factors contributing to the limited clinical success include poor mAb penetration due to biofilm-associated extracellular matrices, which restrict diffusion of large biomolecules, and reliance on host immune effector functions such as complement-mediated clearance and opsonization (64, 65). In clinical settings, pathogen identification should be accomplished promptly to select the mAbs; otherwise, treatments may yield undesirable outcomes. Moreover, developing mAbs requires investment, which is in conflict with the motivation of pharmaceutical companies. As a result, there are only few available mAbs for antimicrobial applications in the market.

### Antimicrobial peptides

The discovery of magainins, isolated from *Xenopus laevis* (African clawed frog) by Michael Zasloff, marked the beginning of research in AMPs (66). However, after 40 years of research into their antibacterial and antifungal potential (67), clinical translation has been limited. This is due to intrinsic clinical challenges, including poor stability and an unfavorable bioavailability profile (68, 69). Nevertheless, continued research in AMPs, together with advances in computational approaches and AI-enabled tools, has contributed to efforts to explore the role of AMPs in addressing AMR.

AMPs offer a promising approach to combating multidrug-resistant (MDR) bacteria (70). These peptides disrupt bacterial membranes and inhibit macromolecular synthesis, making them effective against a wide range of pathogens. In addition to their bactericidal properties, AMPs inhibit biofilm formation by preventing bacterial adhesion and disrupting extracellular polymeric substances, which are key components of biofilm stability. To address MDR, Shi et al. developed peptide LI14 that exhibits broad-spectrum activity against various pathogens including the methicillin-resistant *S. aureus*, vancomycin-resistant *Enterococci*, and carbapenem-resistant Enterobacteriaceae (CRE) (71). When paired with clinically relevant antibiotics, LI14 inhibited multiple MDR bacteria. The combinatorial effects were later validated in a mouse infection model, demonstrating the efficacy of LI14 alone and in combination with antibiotics. In the context of clinical translation, AMPs have demonstrated promising results when administered for topical treatments in trials. For instance, omiganan pentahydrochloride (MX-226), a topical gel, has been tested for catheter-related infections, and it is capable of inhibiting all commonly occurring fungal pathogens (72). In a randomized, controlled, double-blinded, multicenter trial (NCT00563394 and NCT00563433; Table 1), the efficacy of pexiganan, an AMP-based topical cream, was compared with oral antibiotics for the treatment of diabetic foot ulcers (73). Throughout the trial, bacterial resistance to oral antibiotics emerged, while no resistance was observed for patients receiving pexiganan, pointing to a potential effective alternative to traditional antibiotic-driven treatment strategies. To expedite the development of AMPs, AI-based models, including deepAMP and AMP-Designer are specifically developed to design AMPs swiftly and efficiently (74, 75). Notably, deepAMP designed the T2-9 AMP, demonstrating comparable antibacterial efficacy to FDA-approved antibiotics (74). However, the use of AMPs is limited by their high production costs, structural instability, and low bioavailability (76), posing challenges for systemic applications. Production cost is driven up by the long amino acid sequences of AMPs and complex manufacturing processes. Moreover, AMPs are prone to degradation in the presence of proteases, salts, and pH changes. Aside from these disadvantages, AMPs do possess important features, such as diverse targets and high selectivity (69). With advances in AI, digital strategies guiding AMP development may help overcome challenges mentioned above, potentially expanding the role of AMPs in addressing AMR.

### CRISPR-Cas system

The CRISPR-Cas9 system, which remains preclinical, showcases an advancement in combatting AMR by selectively targeting and eliminating resistance genes (77). This genome-editing technology can help resensitize bacteria to antibiotics, offering a new approach to treating infections. In principle, CRISPR-Cas9 knocks out resistance genes from bacterial DNA to prevent resistant mechanisms from functioning and restores the potency of antibiotics. CRISPR-Cas9 has been designed to target resistance genes like *mcr-1*, *blaIMP-1*, and *tetM*, which mediate resistance to colistin, carbapenems, and tetracyclines, respectively (78). A study resensitized *E. coli* to meropenem and colistin by using CRISPR-Cas9, reducing their minimal inhibitory concentrations (MICs) by over 4- and 64-fold, respectively (79). Apart from resensitizing-resistant bacteria to antimicrobials, CRISPR-Cas9 can also impede the transfer of resistance genes between bacteria, thus constraining the spread of drug resistance. For example, a customized CRISPR-Cas9 system successfully protected *E. coli* strains from acquiring resistance genes through horizontal gene transfer (80). The versatility of the CRISPR-Cas9 system may address the AMR crisis through gene editing (80).

### Nanoparticles

One of the common drivers of AMR is chromosomal mutations that alter the targets for existing antimicrobial drugs, thereby decreasing treatment efficacy (81). Nanoparticle-based antibacterial approaches, while still preclinical, have physicochemical properties that enable broad-spectrum antimicrobial activity and can circumvent conventional AMR mechanisms via novel pathways. For example, a titanium oxide–based nanocomposite can penetrate the bacterial membrane with its fiber-like carbon substrate and cause mechanical disruptions in *P. aeruginosa* (82). The titanium oxides further disrupt the electron transport chain via excess electron influx into key proteins, inducing bactericide. Independent of surface chemistry, large quasi-spherical gold nanoparticles wrapped by the membrane create deformations that lead to membrane rupture in *S. aureus* and *P. aeruginosa* (83). The quasi-spherical morphology has a greater interactive affinity compared with a star-shaped morphology. Guided by physics and chemistry principles, predictive and generative AI (GenAI) models can aid in the selection of nanomaterials and the design of particle morphologies to optimize therapeutic strategies against AMR. These strategies include enhancing bactericidal activity, reducing toxicity, increasing the precision of drug delivery to bacterial targets, and controlling drug release.

## Harnessing AI to accelerate drug development

Emerging technologies such as the CRISPR-Cas9 gene editing system have provided alternative solutions to address AMR. However, most of them require additional validation work and fine-tuning prior to large-scale implementation and clinical translation. In this unprecedented arms race against AMR, AI may serve as a critical catalyst to stimulate the innovation of alternative strategies for drug development that complement existing workflows. Even though AI-enabled approaches have supported various aspects of antibiotic discovery and development, their ability to accelerate the overall antibiotic development workflow remains uncertain. Furthermore, major scientific and translational bottlenecks, including the limited availability of novel antibacterial targets, challenges in translating in vitro findings into successful preclinical/clinical outcomes, and regulatory constraints, continue to impact antibiotic development. Therefore, the extent to which AI can accelerate the discovery of clinically effective antibiotics has not been fully understood. More realistically, AI may function as a supportive tool that improves efficiency across various stages of antibiotic discovery, development, and clinical decision support rather than completely transforming the entire research and clinical workflows for AMR. Nonetheless, meaningful progress against AMR will require coordinated global efforts spanning scientists, clinicians, industry partners, regulatory agencies, and policymakers.

### Drug repurposing: IDentif.AI—a digital medicine platform

Existing drug development workflows are costly and time-consuming (84). To sustainably design therapeutics and combat the emergence of AMR, drug repurposing has received attention (85). For example, during the COVID-19 pandemic, repurposed drug remdesivir, an antiviral developed to treat the Ebola virus, quickly became the primary therapeutic for COVID-19 patients (86). Importantly, repurposed drug candidates are more likely to receive FDA approval with fewer risks and shorter review period (85). Drug repurposing has also gained traction for disease indications spanning from cancer to heart disorders (18, 87). This approach has become an important strategy to address the limited development of novel antibiotics (88).

**Figure 2:**
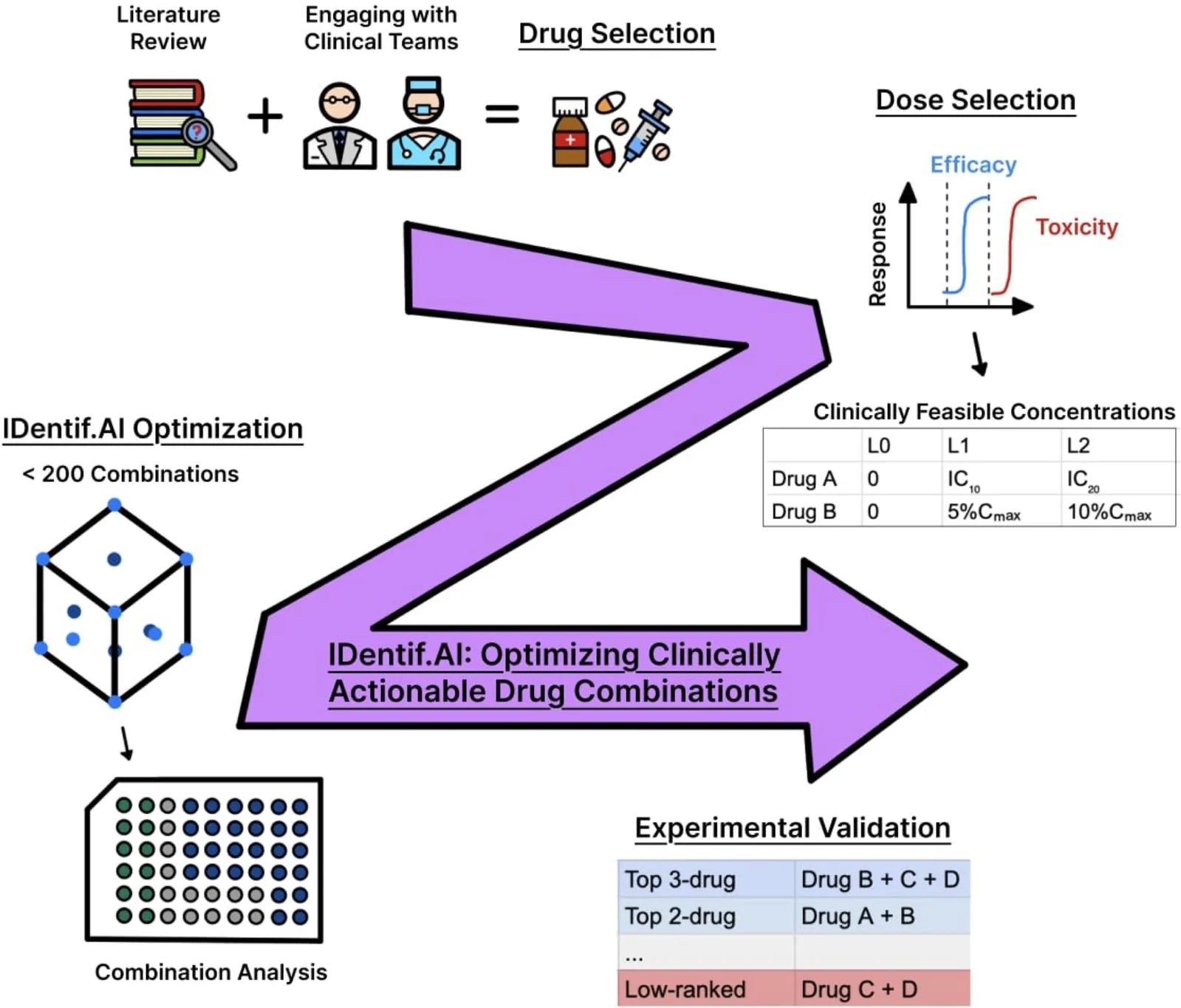
IDentif.AI's community-driven workflow. The workflow begins with screening for potential drug candidates via literature search as well as inputs from clinical teams and infectious disease experts. Subsequently, each of the drug candidates is assessed via dose–response assay to determine monotherapy efficacy. Based on the dose–response curve and pharmacological data from clinical trials, three clinical feasible drug concentrations are selected for IDentif.AI optimization. In the optimization step, a set of curated (<200) drug combinations are experimentally assessed. Correlating the input combinations and their biological response (e.g. %Inhibition) via an AI-discovered relationship, IDentif.AI interrogates the drug–drug interaction space and provides a ranked list of all possible combinations in the parameter space ranking from top to low efficacy. Lastly, clinical teams and experts are actively involved in the selection of clinically relevant drug combinations for subsequent experimental validation, which includes in-depth analysis like synergy assessment.

IDentif.AI, an AI-derived digital medicine platform, was developed to rapidly pinpoint effective drug combinations comprising of repurposed candidates to combat pathogens (19–21). This platform is based on an AI-discovered second-order quadratic series derived from the in vitro “modeling” between cellular responses and drug interventions (89). IDentif.AI is a small data-driven platform that experimentally screens <200 drug combinations without preexisting information and big data (90). After the screening, IDentif.AI correlates the input combinations and measured biological response (e.g. viability and inhibition) via the AI-discovered relationship and utilizes the relationship to predict the efficacy of all possible combinations in the parameter space (3, 4, 20, 21, 91, 92). All IDentif.AI-designed combinations are experimentally validated. The IDentif.AI workflow is community driven and engages with clinical teams and infectious disease experts to shortlist the repurposed drug candidates, ensuring that IDentif.AI-designed combinations are clinically actionable and feasible (Fig. 2).

This community-driven IDentif.AI workflow was adapted to address AMR. IDentif.AI was harnessed to determine drug cocktails for “nontuberculous mycobacteria” (3). The platform determined actionable drug combinations that exhibited greater in vitro efficacy than the standard of care regimens (3). In another study, the platform was deployed to identify broad-spectrum drug combinations against CRE (5). The platform pointed to a broadly effective combination comprising meropenem and bleomycin (5). Combining meropenem, a traditional antibiotic, with bleomycin, a cancer drug, demonstrated effective antibacterial efficacy against *E. coli*, *K. pneumoniae*, and *Enterobacter hormaechei*, as well as their clinical isolates (5). The interactions between the two drugs were strongly synergistic across all species and isolates. Similarly, in a study addressing *A. baumannii*, IDentif.AI also designed a broadly effective and synergistic combination against clinical isolates (4). IDentif.AI has demonstrated its versatility and fast turnaround time, representing an important strategy that may accelerate AMR-specific drug development.

Aside from IDentif.AI, multiple studies have harnessed the power of AI to determine effective repurposed drug candidates to address AMR. Repurposed drug candidates, whether used individually or in combination, may not always be sufficient to counter emerging potent infections. Thus, alternative approaches that complement but adopt a markedly different route to drug combinations (e.g. AI-driven discovery of novel drugs) are warranted. For instance, researchers introduced a machine learning–based strategy to accelerate antibiotic discovery (93). This deep neural network is trained to predict antibacterial activity against *E. coli* using a dataset of 2,335 compounds, including FDA-approved drugs and natural products. The model was applied to screen over 100 million molecules from chemical libraries, including the Drug Repurposing Hub. A key outcome of this screening was the identification of halicin, a preclinical investigational drug for diabetes, and this compound is structurally unrelated to traditional antibiotics. Halicin demonstrated broad-spectrum bactericidal activity, including efficacy against *Mycobacterium tuberculosis*, *C. difficile*, and MDR *A. baumannii* in mouse models. In another study, a machine learning model based on chemical descriptors of FDA-approved and other drugs was harnessed to identify compounds targeting the CaFKS1 synthase of *Candida albicans* (94). AMR is a particular concern among the *Candida* genus, as ∼30% of isolates are resistant to antifungal drugs (94). This model is capable of predicting the efficacy of drugs against the CaFKS1 synthase. Multiple repurposed drugs, including icatibant (used to treat sudden attacks of hereditary angioedema), were identified by the model. Pairing AI-enabled approaches with the concept of repurposing drugs may potentially yield actionable therapeutics against AMR. However, the identification of compounds with antibacterial activity alone does not necessarily translate into clinically actionable and feasible antibiotics. Further validation of AI-identified compounds, such as halicin, particularly regarding its resistance patterns, toxicity profiles, and preclinical and clinical outcomes, may provide more insights into the actionability of these AI-driven approaches and compounds identified through them.

### AI-driven structural predictions

Recently, computational tools have been developed for the purpose of employing machine learning concepts to accurately predict biomolecular structures of compounds with antimicrobial effects. In a relevant study, scientists from the Massachusetts Institute of Technology and Harvard University argued that neural network models can predict structural classes of antibiotics through identifying chemical substructures in association with antibiotic activity. The research team trained graph neural networks on datasets of antibiotic activity and human cell cytotoxicity in 39,312 compounds to predict the antibiotic activity and cytotoxicity for 12,076,365 other compounds. By empirically testing the top compounds, successful results demonstrate that deep learning and machine learning models are explainable and functional for structural predictions in the antibiotic development process (23).

A recent study applied machine learning to discover novel antibiotics targeting *A. baumannii*, a highly drug-resistant gram-negative pathogen associated with hospital-acquired infections (95). The team screened 7,500 molecules for *A. baumannii* growth inhibition and used this dataset along with the structures of each molecule to train a message-passing neural network. The model was applied to predict structurally novel compounds with activity against the pathogen. Through this approach, the team identified abaucin, an AI-discovered compound with narrow-spectrum antibacterial activity against *A. baumannii*. Abaucin was shown to disrupt lipoprotein trafficking by targeting LolE, a protein involved in transporting lipoproteins to the outer membrane. The compound demonstrated efficacy in a mouse wound infection model. However, the resistance frequency of abaucin (10^−7^ to 10^−8^) may lead to the emergence of resistant subpopulations. The relatively low inoculum used in the wound infection model may not reveal such resistance. Furthermore, a team harnessed computational screening to identify novel AMPs encoded within the human microbiome. Torres et al. analyzed over 444,000 small open reading frames from 1,773 human metagenomes, predicting antimicrobial activity for 323 peptide candidates (96). Several peptides targeted bacterial membranes, worked synergistically and showed selective activity against pathogens while sparing commensals. The emergence of computational tools in biomolecular structural prediction, including Google DeepMind's AlphaFold and the aforementioned AI models, demonstrates immense potential for the hastening of both biomolecular structure and the antibiotics discovery process (97–99).

### AI-assisted molecular generation

A new frontier of AI-related efforts against AMR is de novo drug design. De novo drug design refers to the computational process of creating novel compounds with desirable properties entirely from scratch without the reliance on existing natural compounds (100). The chemical space is explored by using generative models to generate compounds, but limitations, such as the inability to synthesize generated compounds, persist. In recent years, differing generative modeling approaches have been used as part of the drug discovery process for the purpose of generating desirable molecular compounds, including variational autoencoders (VAEs), generative adversarial networks (GANs), and diffusion models. In a VAE architecture, during the training phase, the encoder receives input data, processes it into a probability distribution in a latent space, to which the decoder uses to reconstruct the original data. For generation, the decoder uses the probability distribution from the latent space to generate new and coherent data (101). The generalizability of this architecture may assist with de novo drug design. For example, DeepICL, developed by the Korea Advanced Institute of Science and Technology, generates ligands with favorable interactions in the context of drug discovery (102). Furthermore, other research teams have applied GAN and diffusion models for the purposes of de novo drug design (103). Instead of creating molecular compounds from scratch, diffusion models implemented in tools like DiffSBDD allow for fixed restraints that generate synthesizable and practical compounds (104). While each feature differs processes and uses, diffusion models, GANs, and VAEs are all deep learning architectures used in various recent approaches to tackling molecular compound generation. By exploring chemical spaces and generating new compounds without the restraints of working with existing compounds, AI innovates drug discovery in the frontier of de novo drug design (105).

### Generative AI and other AI-powered solutions

GenAI has been harnessed to support AMR-specific drug development (106, 107). In a study, SyntheMol, a GenAI-driven platform, was deployed to design easily synthesizable, structurally novel antibiotics against *A. baumannii* (106). This framework begins by experimentally screening three chemical libraries against *A. baumannii* (ATCC17978) to generate a training dataset. Following incubation, the OD_600_ of each compound was measured to assess their antibacterial activity. From these results, the study team classified active and inactive compounds from the initial pool. Using these prospective data, the team trained three property prediction models to predict antibacterial activity against *A. baumannii*. SyntheMol utilized the prediction models together with Monte Carlo Tree Search to iteratively assemble molecules from around 132,000 validated chemical building blocks and 13 known chemical reactions. This enabled the platform to explore the chemical parameter space containing more than 30 billion possible compounds. The platform identified six structurally novel antibiotics that demonstrate antibacterial activity against the bacteria. The whole GenAI-driven workflow can be done in 3 months.

Beyond predicting structures and generating molecular compounds, AI may be used to predict synergy interactions among drug candidates in the antibiotic discovery process (108). For example, machine learning methods like graph learning can be used for synergy prediction. In a 2022 study, Lv et al. collected drug–target interactions from literature, along with a graph learning framework to predict potential synergistic antibiotic combinations (109). This study found that synergistic antibiotic combinations have overlapping patterns and specific network topological relationships. By feeding the network proximity of the discovered drug pairs into a graph regularization model, they were able to predict new synergistic antibiotic combinations. Another study incorporated machine learning in synergy prediction for AMPs and antimicrobial agents (110). The most efficient machine learning classifier was able to achieve 76.92% in predicting synergistic combinations, enabling rapid and efficient combinatorial design without overutilizing laboratory resources. Nonetheless, these approaches primarily identify combinatorial strategies that may improve antibacterial efficacy. However, in the context of clinical actionability, the ability of these combinations to substantially reduce resistance frequencies is also crucial. Therefore, when designing synergistic drug combinations, both therapeutic efficacy and resistance patterns should be considered.

### Aligning AI-enabled solutions with global communities

While AI-enabled approaches have demonstrated potential in advancing AMR-centric therapeutic development, decision support, and pandemic readiness, technological innovation alone is insufficient to effectively contain the emergence of AMR. For instance, the rapid expansion in GenAI has enabled multiple solutions to AMR beyond drug development including antibiotic prescription and public health/educational interventions (111–116). However, the successful implementation of these advanced technologies into real-world workflows for addressing AMR requires active and coordinated global collaboration. In the context of global public health, AI should not be viewed as the final solution to AMR; instead, it serves as a key catalyst that enables further collaborative efforts to address this global challenge. This community-driven framework may efficiently align global strategies in educational/public health initiatives, governance, policy, and other relevant considerations, promoting a collaborative approach to effectively address AMR.

## A community-driven approach to address AMR

**Figure 3:**
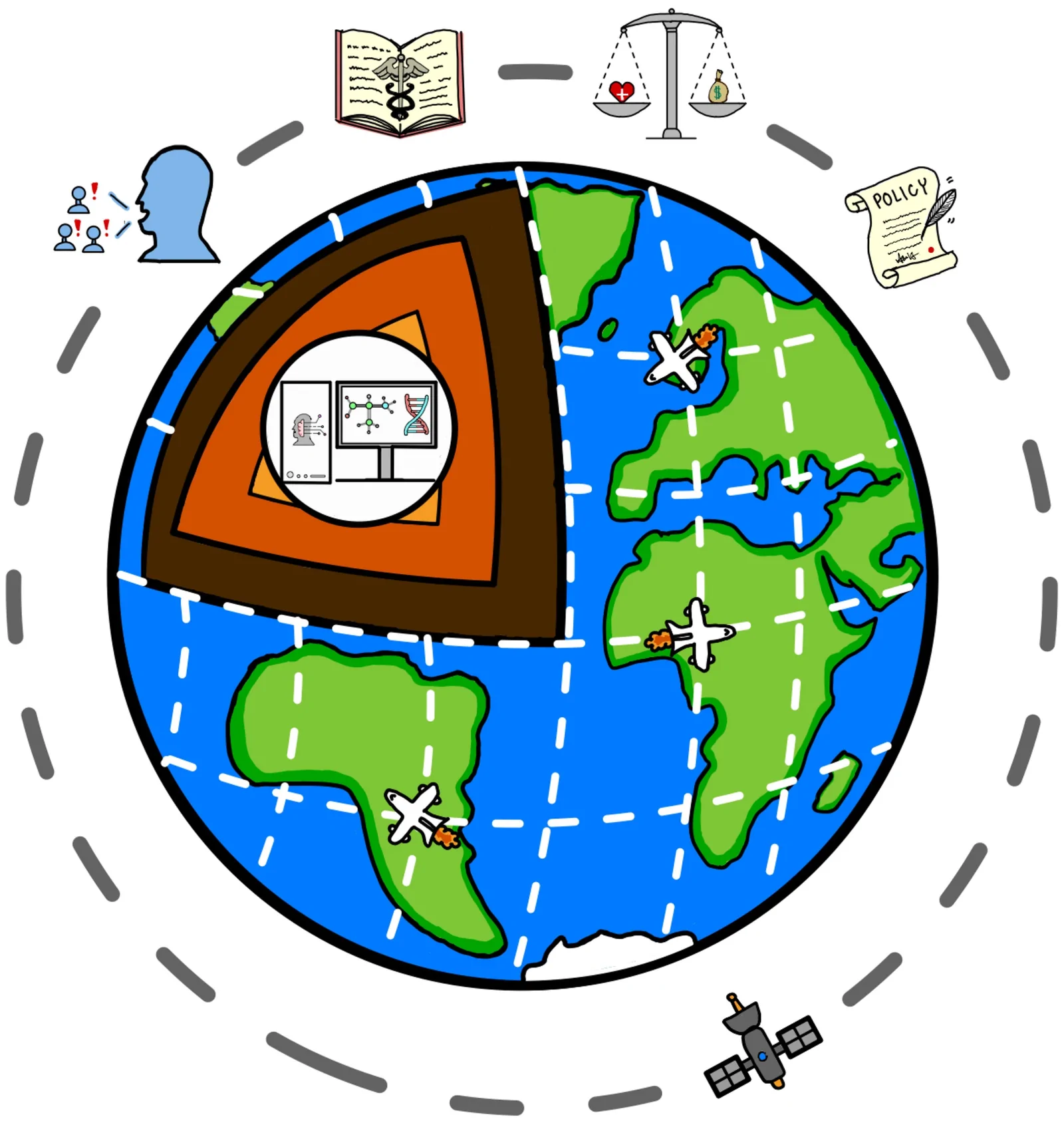
An inclusive, community-driven approach to address AMR. Beyond integrating AI and machine learning into the drug discovery process, inclusive community-driven strategies, including public awareness initiatives, antimicrobial stewardship programs, health economics, policymaking, and coordinated stakeholder engagement, are also essential for alleviating the global threat of AMR.

Previous discussions have highlighted the challenges associated with antibiotic development and reviewed AI-powered platforms that may alleviate the emergence of AMR. However, beyond drug development, broader considerations including health economics and incentives for antibiotic innovation should also be carefully addressed. Building on the perspective that AI is a catalyst toward collaborative efforts, this section proposes a community-driven framework to overcome AMR-associated challenges through engagement with diverse stakeholders and communities including scientists, health economists, policymakers, and public health experts (Fig. 3). Notably, community-based HIV initiatives contributed to the Joint United Nations Programme on HIV/AIDS (UNAIDS) 90-90-90 targets that aimed to end the HIV epidemic. Community-driven efforts through health workers, peer workers, and educational/technological interventions directly contributed to the UNAIDS targets (117). Building on this precedent, a tailored community-driven framework may lead to effective, sustainable strategies to address AMR. However, it is important to note that the success of the UNAIDS framework was supported by active communications among stakeholders globally. While the proposed AI-powered, community-driven workflow may present inherent operational challenges, prioritizing structured and sustained global communication to facilitate data sharing, coordination, and alignment across stakeholders represents a critical first step toward scalable implementation of this framework.

### Educational and public health interventions

An adaptive health communication strategy among the general public, clinicians, and targeted groups is one of the main pillars in the fight against AMR. Its primary purpose is to reduce antibiotic consumption by raising awareness of the negative consequences of excess use. In general, such strategies employ a mixture of behavioral mechanisms to foster improved decision-making. Approximately 80% of the campaigns target the general public and physicians (118), highlighting the importance of coordinated engagement across stakeholders.

The knowledge channel is adopted in public health communication to improve public understanding of antibiotics. This mechanism is necessary, for example, a 2024 survey revealed that only 34–77% of respondents from different European Union countries recognized that antibiotics are ineffective against viral infections (119). A systematic review of intervention programs shows that five out of eight general programs significantly improved the patients' understanding of proper antibiotic use. Stewardship behavior also improved in the same studies measured by the prevalence of misuse and interrupted therapy. The remaining three programs did not have a significant impact on the outcome measures (120). These findings highlight the potential benefit and uncertainty of educational interventions, further accentuating the need for approaches that are not only informative but also consistently actionable in real-world settings.

AI and machine learning have emerged as transformative approaches in the development of digital tools to combat AMR, particularly within the context of inherently complex decision-making processes. For example, prescribing antibiotics requires clinicians to integrate multiple factors: patient-specific conditions, suspected/confirmed pathogens, resistance patterns, treatment guidelines, and among many other considerations. Thus, it is important to recognize that developing successful AI-enabled tools requires continuous engagement with clinicians (users), who provide feedback including operational integration, user interface/experience design, behavioral considerations, and among others (92). Successful implementation depends on integrating these AI-enabled tools seamlessly within existing clinical workflows to support timely and patient-specific decision-making without further administrative burden. In parallel, improving the digital literacy of clinical teams remains essential to ensure appropriate interpretation and utilization of AI-driven clinical decision support in clinical settings (121). For instance, AI-powered prescription support tools are increasingly integrated into ASPs to assist clinicians in diagnosis and antibiotic selection, thereby reducing the likelihood of overuse and misuse (122, 123). In this context, AI is not intended to replace clinical judgment but to provide assistance to clinical decision-making based on coordinated communications among global stakeholders.

While integrating AI-enabled tools into clinical workflows is essential for improved clinical decision-making, simultaneous efforts at the population level are also critical to drive proper antibiotic use. Beyond clinical applications, GenAI and large language models offer valuable support for public health education by enhancing awareness and understanding of AMR through multiple avenues. GenAI can create engaging educational videos, infographics, or interactive quizzes that simplify complex information and improve comprehension. Importantly, GenAI tools can be used to generate tailored content for different educational levels, age groups, and languages, and support gamified learning through interactive games and simulations. When integrated into public health strategies, these approaches may improve accessibility, retention, and actionable AMR-related knowledge (124).

Though GenAI holds promise for scaling AMR education, it presents challenges when used uncritically, particularly regarding accuracy, contextual relevance, hallucinated content, and responsibility for errors arising from AI use (123, 125). Therefore, the most effective approach is using GenAI as a tool for generating initial drafts, summaries, or visuals, and domain experts ensure content accuracy, relevance, and appropriateness through careful review and refinement. This expert-guided oversight of GenAI-generated contents is essential to ensure accountability, accuracy, and consistency across clinical and public health contexts. These educational and public health interventions across clinical teams and the general public highlight the role of AI across healthcare settings, with alignment to workflows to ensure sustained impact in addressing AMR challenges.

### Governance and policy

In response to AMR, institutionally and government-led programs, including those that are cross-systems have been established to provide systematic surveillance to monitor resistance trends. For example, in 2015, WHO Member States approved the Global Action Plan to Tackle AMR (GAP-AMR) program, and in the same year, launched the Global Antimicrobial Resistance and Use Surveillance System (GLASS) to bridge and standardize global surveillance initiatives (126). Furthermore, GLASS promotes a collaborative approach for data sharing that assesses resistance trends in epidemiological, clinical, and population-level data, aside from conventional laboratory-based datasets. Aside from these global programs, nations have also established their own independent institutes to promote governance and surveillance over AMR (127). For example, the US government has established a program known as the National Antimicrobial Resistance Monitoring System (NARMS), a joint program by the FDA, CDC, and United States Department of Agriculture, with surveillance spanning from people to food animals (128). Meanwhile, the Singapore Ministry of Health summarizes surveillance findings in public reports for community members (e.g. public health official, clinicians, and infectious disease experts) to reference and review (129). Though programs like GAP-AMR, GLASS, and NARMS have promoted surveillance nation-wide and on a global scale, considerations including (i) misrepresenting current trends using laboratory samples, (ii) failing to effectively bridge human and food-animal surveillance, and (iii) effective communications among international members are critical to reduce the burden of AMR (130).

Furthermore, providing guidance on the proper use of antibiotics to prevent overuse and misuse is also critical to alleviate the threat of AMR. For example, overprescribing antibiotics in primary care is a major concern, and >90% of antibiotics are prescribed for respiratory tract infections by general practitioners (17). According to CDC, over 30% antibiotics prescribed in the United States are deemed unnecessary (37). To prevent misuse and overuse of antibiotics, government-led programs like ASPs have intervened to provide guidelines on antimicrobial usage in order to improve patient outcomes, reduce resistance, and decrease healthcare costs (131, 132). A study has pointed to the substantial reduction of antibiotic usage globally as a result of implementing ASPs (133). Therefore, promoting governance through government-led programs may serve as a critical strategy for combatting AMR. However, open access to data and discussions as well as policies facilitating communications among international members are also critically important to overcoming challenges associated with AMR.

In addition to government-led programs, policies pertaining to antibiotic usage in food chains should also be carefully reviewed. Notably, the uses of antibiotics in food chain have made food animals one of the major reservoirs of resistant bacteria (134). The One Health framework has been implemented to promote cross-system communications—human/animal health, agriculture, and environmental science—to collaboratively address surveillance, guidance, and stewardship toward responsible antibiotic applications across all sectors (135, 136). Furthermore, these policies and surveillance/stewardship programs should also be enforced in lower-to-mid-income regions, where animal–human interactions may result in high-risk transmissions of resistant pathogens. Previous studies have also emphasized that government initiatives toward reducing poverty along with different policies and frameworks may be the most effective in curtailing the emergence of AMR (135).

### Economic incentives for innovation

Both scientific literature and industry commentary recognize that the lack of investment in antibiotic development stems from poor market incentives. The number of large pharmaceutical companies with an active antibiotic R&D pipeline dropped from 18 in 1990 to only six in 2016 (137). Paradoxically, antibiotics attract a sizable market, but the profit margins are too low compared with the sunk costs of R&D. By the end of the clinical trials, the developer faces an expected cumulative investment cost of $1.5 billion (138). The nature of antibiotic therapy limits demand. They are typically prescribed for short courses, limiting revenue.

The timeframe for antibiotics fails to encourage investment. Although patent protection typically lasts for 20 years, the patent filing takes place before clinical trials. Estimates vary, but this delay decreases the market exclusivity of a new drug to only 8–12 years (139). However, it takes ∼10 years to break even (140). After patent expiration, generic products sharply reduce profit, a phenomenon coined as patent cliff. In addition to the unfavorable projected numbers, pharmaceutical companies also face substantial financial risk. The success rate of moving from the preclinical research stage to achieving regulatory approval ranges between 1.5 and 3.5% (141).

Several solutions have been proposed by decision-makers to improve incentives to invest in antibiotics development. Most ideas focus on push—subsidy-oriented methods, including direct funding and tax benefits—and pull—reward-oriented methods including entry reward or subscription models—or a mix of these pathways. Ongoing government programs test these approaches, but their impact is yet to be seen (142, 143).

### Health economics considerations

The global threat posed by AMR extends beyond public health to encompass economic implications. The World Bank projects that AMR could result in $1 trillion in additional healthcare costs by 2050, alongside $1 trillion to $3.4 trillion in gross domestic product (GDP) losses per year by 2030 (144). These economic impacts manifest through interconnected pathways: prolonged hospital stays for resistant infections, costly second- and third-line treatments when first-line antibiotics prove ineffective, as well as lost productivity from extended sick leave and increased mortality rates. The relationship between AMR and increased mortality, morbidity, length of hospitalization, and cost of healthcare has been well established in the literature (145).

Empirical evidence demonstrates the burden that resistant infections place on healthcare systems. A comprehensive analysis of 73,244 patients with infection-related hospitalizations revealed that 15.9% had AMR infections, with these patients experiencing significantly longer cumulative length of hospital stays, reaching 20.4 days and 2.9 additional days for skin infections (146). The economic burden extends beyond the initial hospitalization, as approximately half of the patients admitted with AMR infections required readmission within the subsequent year, further amplifying healthcare costs and system strain.

In low- and middle-income countries (LMICs), several structural factors amplify AMR's economic impact. Weaker healthcare systems with limited diagnostic capacity lead to reliance on empirical antibiotic use, accelerating resistance development. Limited access to newer, more expensive antibiotics means that when first-line drugs fail, treatment options become prohibitively costly or unavailable. Higher baseline infectious disease burden in these settings means AMR affects a larger proportion of the population. The predominance of out-of-pocket healthcare payments in LMICs means families face catastrophic expenses when seeking treatment for resistant infections. Macroeconomic evidence shows that AMR is not just a health threat, but a powerful driver of economic divergence as LMICs face steeper challenges. Estimates show that low-income economies' GDP may contract by about 5.6% by 2050. They will not fare better at healthcare spending, which may have to rise by roughly 25% due to AMR (147). These changes translate into welfare losses: as many as 28 million additional people could be pushed into extreme poverty, most of them in LMICs. Echoing these findings, the WHO emphasizes that the drivers and consequences of AMR are exacerbated by poverty and inequality, threatening progress toward the Sustainable Development Goals (8).

## Conclusion

In this review article, challenges in addressing AMR, including regulatory constraints, scientific bottlenecks, and the lack of incentives for AMR-specific drug development, are discussed. Aside from traditional antibiotic-oriented treatment, other strategies, such as bacteriophages and CRISPR-Cas9, have complemented conventional standards of care with promising results in some real-world use cases. Notably, the AI-driven structural prediction modality yielded a novel drug with potent activity against the ESKAPE pathogens. With advances in AI, AI-enabled approaches may further improve efficiency in multiple stages of antibiotic discovery and development when integrated with global efforts to address scientific and translational challenges. However, reliance on AI-driven approaches alone is unlikely to be sufficient to overcome global challenges associated with AMR. An inclusive, collaborative, community-driven approach engaging with stakeholders (e.g. policymakers, health economics experts, government-led programs, public health officials, etc.) may potentially alleviate the global threats posed by emerging AMR pathogens. Continuous technological advancement, as well as effective, collaborative communications across all communities, are the foundation of properly addressing AMR.
